# Telomere length in breast cancer patients before and after chemotherapy with or without stem cell transplantation

**DOI:** 10.1054/bjoc.2001.1803

**Published:** 2001-05

**Authors:** C P Schröder, G B A Wisman, S de Jong, W T A van der Graaf, M H J Ruiters, N H Mulder, L F M H de Leij, A G J van der Zee, E G E de Vries

**Affiliations:** Departments of Medical Oncology, Gynecologic Oncology, Pathology and Laboratory Medicine, University Hospital Groningen, The Netherlands

**Keywords:** breast cancer, telomere length, high-dose chemotherapy, autologous stem cell transplantation

## Abstract

High-dose chemotherapy and peripheral blood stem cell transplantation (PBSCT) may accelerate telomere length loss in haematopoietic stem cells. As data including pre-and post-treatment samples are lacking, we studied leukocyte telomere length and telomerase activity before and after treatment in breast cancer patients randomized to receive 5 adjuvant courses FEC (5-FU, epirubicin and cyclophosphamide) (*n*= 17), or 4 × FEC followed by high-dose cyclophosphamide, thiotepa, carboplatin and autologous PBSCT (*n*= 16). Haemoglobin, MCV, leukocyte-and platelet numbers were assessed prior to (*t*_0_), 5 months after (*t*_1_) and 9 months after chemotherapy (*t*_2_); these parameters were decreased at *t*_1_ and *t*_2_ compared to *t*_0_(high-dose: all parameters; standard-dose: leukocytes and platelets), and all parameters were lower after high-dose than standard-dose treatment at *t*_1_. Paired individual leukocyte samples of *t*_0_ and *t*_1_ showed telomere length change (determined by telomere restricted fragment (TRF) assay) ranging from +0.8 to –2.2 kb, with a decreased TRF length in 9 patients of both groups. Telomerase activity (determined by TRAP assay) was below detection limit in leukocyte samples of *t*_0_ and *t*_1_. Thus, standard-and high-dose chemotherapy negatively affect haematological reconstitution in this setting. In individual patients, telomere length can be remarkably changed following haematological proliferative stress after treatment. © 2001 Cancer Research Campaign www.bjcancer.com


*British Journal of Cancer* (2001) **84**(10), 1348–1353
© 2001 Cancer Research Campaign
doi: 10.1054/ bjoc.2001.1803, available online at http://www.idealibrary.com on IDEAL®

### Telomere length in breast cancer patients before and after chemotherapy with or without stem cell transplantation

**CP Schröder[1], GBA Wisman[2], S de Jong[3], WTA van der Graaf[1], MHJ Ruiters[3], NH Mulder[1], LFMH de Leij[3], AGJ van der Zee[2] and EGE de Vries[1]**

Departments of Medical Oncology[1], Gynecologic Oncology[2], Pathology and Laboratory Medicine[3], University Hospital Groningen, The Netherlands

**Summary** High-dose chemotherapy and peripheral blood stem cell transplantation (PBSCT) may accelerate telomere length loss in haematopoietic stem cells. As data including pre-and post-treatment samples are lacking, we studied leukocyte telomere length and telomerase activity before and after treatment in breast cancer patients randomized to receive 5 adjuvant courses FEC (5-FU, epirubicin and cyclophosphamide) ($n$ = 17), or 4 × FEC followed by high-dose cyclophosphamide, thiotepa, carboplatin and autologous PBSCT ($n$ = 16). Haemoglobin, MCV, leukocyte-and platelet numbers were assessed prior to ($t_0$), 5 months after ($t_1$) and 9 months after chemotherapy ($t_2$); these parameters were decreased at $t_1$ and $t_2$ compared to $t_0$ (high-dose: all parameters; standard-dose: leukocytes and platelets), and all parameters were lower after high-dose than standard-dose treatment at $t_1$. Paired individual leukocyte samples of $t_0$ and $t_1$ showed telomere length change (determined by telomere restricted fragment (TRF) assay) ranging from +0.8 to –2.2 kb, with a decreased TRF length in 9 patients of both groups. Telomerase activity (determined by TRAP assay) was below detection limit in leukocyte samples of $t_0$ and $t_1$. Thus, standard-and high-dose chemotherapy negatively affect haematological reconstitution in this setting. In individual patients, telomere length can be remarkably changed following haematological proliferative stress after treatment. © 2001 Cancer Research Campaign http://www.bjcancer.com

**Keywords**: breast cancer; telomere length; high-dose chemotherapy; autologous stem cell transplantation

Human telomeres are regions at the chromosomal ends that play an important role in the structure and function of chromosomes. In normal somatic cells telomeres are shortened with every cell division, and when a critical size is reached, cells lose their proliferative potential (Harley et al, 1990; Hastie et al, 1990; Harley, 1997). Also in purified haematopoietic stem cells telomeric DNA appears to shorten with each cell division and thus with age (Vaziri et al, 1994; Lansdorp, 1995). A number of studies have indicated a possible accelerated shortening of telomere length in haematopoietic stem cells, due to proliferative stress following peripheral blood stem cell transplantation (PBSCT) (Shapiro et al, 1996; Notaro et al, 1997; Akiyama et al, 1998; Ball et al, 1998; Wynn et al, 1998; Lee et al, 1999; Akiyama et al, 2000). Although low levels of telomerase- a ribonucleoprotein that synthesizes telomeric DNA- can be determined in CD34+ haematopoietic stem cells, this appears to be insufficient to compensate increased shortening of telomere length (Notaro et al, 1997). Because of possible negative long-term effects of this shortening, including possible cytogenetic abnormalities (Ball et al, 1998; Ohyashiki et al, 1999), genomic instability preceding myelodysplastic syndromes (Ohyashiki et al, 1994) and reduced response following haematopoietic stress (Rudolph et al, 1999), this is clearly of clinical interest. However, most data so far are obtained from allogenetic transplantation settings (Notaro et al, 1997; Ball et al, 1998; Wynn et al, 1998; Lee et al, 1999; Akiyama et al, 2000), in paediatric patients. Fewer data are available on the effect of autologous PBSCT (Shapiro et al, 1996; Akiyama et al, 1998; Lee et al, 1999; Akiyama et al, 2000), while paired data including pre-treatment samples are lacking.

As telomere length of nucleated blood cells was shown to be widely variable between age-matched individuals (Notaro et al, 1997; Akiyama et al, 1998; Ball et al, 1998; Wynn et al, 1998; Lee et al, 1999), prospective paired data are essential for determining the impact of autologous PBSCT on this possible ageing process. Therefore, we prospectively studied leukocyte telomere length and telomerase activity in a group of high-risk breast cancer patients randomized to receive either adjuvant standard-dose chemotherapy, or adjuvant high-dose chemotherapy and PBSCT (De Vries et al, 1996). Paired samples before and after these treatments were compared, allowing assessment of the impact of standard and high-dose chemotherapy on telomere length and telomerase activity.

### PATIENTS AND METHODS

### Patients

Patients included in this study participated in a national randomized adjuvant breast carcinoma study (De Vries et al, 1996). Chemotherapy naive breast cancer patients with 4 or more tumour-involved axillary lymph nodes (stage II and III), ≤55 years of age with negative chest X-ray, liver ultrasound and bone scan, were randomized to receive 5 courses of standard-dose chemotherapy

*Received 25 October 2000*
*Revised 22 February 2001*
*Accepted 28 February 2001*

*Correspondence to:* EGE de Vries

CP Schröder and GBA Wisman contributed equally to this work. This study was supported by grants RUG 96-1277 and 97-1581 of the Dutch Cancer Society.

followed by radiotherapy, or 4 courses of the same combination chemotherapy followed by high-dose chemotherapy, PBSCT and radiotherapy. These groups will be referred to as the standard-dose group, and the high-dose group, respectively. The combination chemotherapy consisted of 5-fluorouracil (500 mg $m^{-2}$), epirubicin (90 mg $m^{-2}$) and cyclophosphamide (500 mg $m^{-2}$), administered intravenously once every 3 weeks. For the high-dose group, PBSC were mobilized following the third or last course of FEC with daily subcutaneous recombinant human granulocyte-colony stimulating growth factor (rhG-CSF, 263 µg), from day 2 of the course. Leucapheresis was performed from day 9 of this course, until ≥$5.10^6$ CD34+ cells $kg^{-1}$ body weight (as determined by flow cytometric analysis with the fluorescein isothiocyanate-labelled anti-CD34 antibody directed against the HPCA-2 epitope on CD34+ cells, Becton Dickinson, Leiden, the Netherlands) were obtained. High-dose chemotherapy consisted of cyclophosphamide (1500 mg $m^{-2}$), thiotepa (120 mg $m^{-2}$) and carboplatin (400 mg $m^{-2}$) on days -6, -5, -4 and -3, followed by reinfusion of PBSC on day 0. After reinfusion, daily subcutaneous rhG-CSF was administered until the leukocyte count exceeded $3.10^9$ $l^{-1}$. Locoregional radiotherapy (50 Gy in 25 fractions) was administered after completion of the chemotherapy scheme with sufficient bone marrow recovery (defined as platelets >$100.10^9$ $l^{-1}$). Oral tamoxifen 40 mg daily was administered after platelet recovery for 2 years, in both groups. The study, and the collection of blood samples as described, was approved by the Medical Ethical Committee of the University Hospital Groningen. All patients gave informed consent.

### Sampling times

Blood samples were collected from all consecutive patients randomized in this study from May 1997 until January 1999. Sampling times were: $t_0$: directly prior to start of chemotherapy; $t_1$: 5 months after completion of chemotherapy; $t_2$: 9 months after completion of chemotherapy.

Telomere length was measured in samples from $t_0$ and $t_1$. In a number of these samples it was also possible to measure telomerase activity. Haematological examinations, e.g. haemoglobin, mean corpuscular volume (MCV), leukocytes, and platelets were performed at $t_0$, $t_1$ as well as $t_2$. Haematological parameters were considered normal with haemoglobin ≥ 7.45 mmol $l^{-1}$, MCV 80–96 fl, leukocytes ≥ $4.0.10^9$ $l^{-1}$ and platelets ≥ $150.10^9$ $l^{-1}$ (Barbui et al, 1996).

### Analysis of telomere length

In blood samples from $t_0$ and $t_1$, lysis of erythrocytes was performed with an ammonium chloride solution (155 mM $NH_4Cl$, 10 mM potassium hydrogen carbonate, 0.1 mM sodium ethylene diamine tetra acetate, EDTA). The remaining nucleated cell fraction was then washed in phosphate buffered saline (PBS) solution (0.14 M NaCl, 2.7 mM KCl, 6.4 mM $Na_2HPO_4.2H_2O$, 1.5 mM $KH_2PO_4$, pH 7.4). After centrifugation (150 ***g***, for 10 min) the supernatant was decanted and $1.10^6$ cells of the pellet of nucleated cells were transferred onto a slide for assessment of the leukocyte differentiation. The remaining pellet of nucleated cells was stored at –80˚C. This erythrocyte lysis procedure was performed in accordance with Wynn et al (1998).

In the nucleated leukocyte cell fraction, mean telomere length was determined by the terminal restriction fragment (TRF) assay according to Harley et al (1990), with minor modifications. DNA was isolated using the salt extraction method as described by Miller et al (1988). 5 µg DNA was digested overnight at 37˚C using 20 U *Rsa*I and 20 U *Hin*fI (Roche Diagnostics, Almere, The Netherlands). Digested DNA was electrophorexed in a 0.6% agarose gel in 0.5× Tris-borate EDTA buffer overnight at 50 V. DNA was depurinated with 0.25 M HCl, denatured in 0.5 M NaOH and 1.5 M NaCl and neutralized in 0.5 M Tris/HCl (pH = 7.5) and 1.5 M NaCl, after which the DNA was transferred to a positively charged nylon membrane (Roche Diagnostics, Almere, The Netherlands) using 10× SSC overnight and dried for 2 hours at 80˚C. Prehybridization, hybridization with 5 ng $ml^{-1}$ probe and washing were performed according to TeloQuant assay (PharMingen, San Diego). Preincubation, incubation with 1:5000 alkaline phosphatase conjugated streptavidine and washing were performed according to biotin luminescence detection kit instructions (Roche Diagnostics, Almere, The Netherlands). Telomeric smears were visualized by incubation of the membrane with the chemiluminescence substrate CPD-Star (1:100), according to the supplied instructions (Tropix, Westburg, Leusden, the Netherlands) and exposure to a film. Films were analysed using a scanner and Diversity One PDI computer software (Pharmacia Biotech, Roosendaal, the Netherlands). The mean TRF lengths were calculated with the formula: mean TRF length = $\sum(OD_i)/\sum(OD_i/L_i)$, in which $OD_i$ is the density output and $L_i$ is the length of the DNA at row i (normally a Gaussian curve was obtained) (Wynn et al, 1998). For standardization, DNA isolated from leukocytes (one sample) of one healthy donor was included on all gels. The mean TRF length of the leukocytes of the donor was 7.3 kb. TRF lengths from patient samples were normalized to the TRF length of the healthy donor sample, which was set at 7.3 kb for each gel analysed. Paired patient samples were always analysed on the same gel. The intra-assay variance coefficient in this study was determined to be 1.4% (95% CI) after analysis of 10 aliquots of the control healthy donor sample on one gel, resulting in a mean measurement variance for each sample of ± 100 bp. Therefore, samples of individual patients with a difference in TRF value < 0.2 kb were considered equal. As control, the plasmid pTSK8 (linearized with *Kpnl*; a kind gift from Dr Royle, Leicester, UK) was used, which contains approximately 200 base pairs (bp) of TTAGGG repeats (Royle et al, 1992). TRF length change (Δ TRF length) was defined as the TRF value at $t_1$ minus the value at $t_0$.

### Telomerase activity (the TRAP assay)

After obtaining the nucleated leukocyte cell fraction as described above, $1.10^6$ leukocytes per telomerase activity assay were lysed in 100 µl TRAP lysis buffer (0.5% CHAPS; 10 mM Tris/HCl (pH7.5); 1 mM $MgCl_2$; 1 mM EGTA; 10% glycerol; 5 mM β-mercaptoethanol; 0.1 mM PMSF) and incubated on ice for 25 min. After centrifugation at 15 000 ***g*** for 20 min at 4˚C, the supernatant was quickly frozen in liquid nitrogen and stored at –80˚C until further processing.

The TRAP assay was performed as previously described (Wisman et al, 1998). In short, telomerase activity levels in leukocytes were determined with a fluorescence-based telomeric repeat amplification protocol assay using $GLC_4$ cells (Zijlstra et al, 1987) as standard in each assay. Peaks representing telomerase activity in $GLC_4$ cell equivalents were summed, then relatively expressed to telomerase activity of 100 $GLC_4$ cell equivalents (set at 100%) and normalized to the signal of modified-internal telomerase assay

© 2001 Cancer Research Campaign standard (M-ITAS). For the samples ($1.10^5$ cells and $1.10^4$ cells) the peaks representing telomerase activity were also summed and normalized to the signal of M-ITAS, thereafter the relative telomerase activity of the leukocytes was correlated to $GLC_4$ cell number (relative quantification comparable to 10 $GLC_4$ cell equivalents = 10 U).

### Statistics

Mean TRF length and telomerase activity in blood samples from in individual patients were compared from $t_0$ and $t_1$, and statistically analysed with the Wilcoxon signed ranks test for paired samples. Comparisons of haematological parameters and leukocyte differentiations between the standard-and high-dose groups, were performed with the Student's *t*-test for independent samples, and comparisons between time points in both groups were performed with the *t*-test for paired samples. Correlations between TRF data, telomerase activity, numbers of CD34+ cells and haematological examinations were examined with the Pearson correlation test. All analyses were performed using the statistical analysis program SPSS. A $P < 0.05$ was considered statistically significant.

### RESULTS

### Patients

The standard-dose group consisted of 17 patients, and the high-dose group of 16. Mean age at the start of treatment was 44.0 years (range 29–54 years) and 44.6 years (range 37–54 years) in these groups respectively (NS). Mean period between blood samples of $t_0$ and $t_1$ was 32 weeks in the standard-dose group, and 37 weeks in the high-dose group (NS).

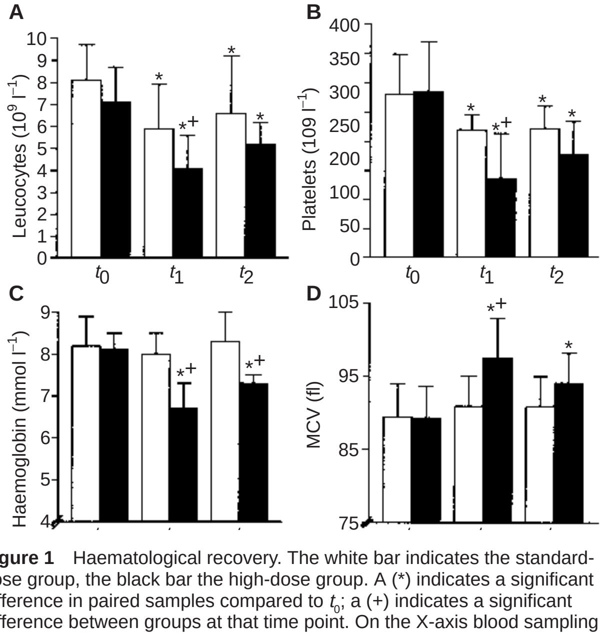

**Figure 1** Haematological recovery. The white bar indicates the standard-dose group, the black bar the high-dose group. A (*) indicates a significant difference in paired samples compared to $t_0$; a (+) indicates a significant difference between groups at that time point. On the X-axis blood sampling times $t_0$ (prior to chemotherapy), $t_1$ (5 months after chemotherapy) and $t_2$ (9 months after chemotherapy) are indicated. On the Y-axis, the following values are indicated with mean + SD: (**A**) leukocytes ($10^9$ $1^{-1}$); (**B**) platelets ($10^9$ $1^{-1}$); (**C**) haemoglobin (mmol $1^{-1}$) and (**D**) MCV (fl)

### Haematological parameters

The analysis of haematological parameters is reflected in Figure 1.

### *Leukocytes*

Compared to the standard-dose group, leukocyte counts were lower in the high-dose group at $t_1$ (standard-dose: mean at $t_1$ $5.9.10^9 l^{-1}$, high-dose: mean $4.1.10^9 l^{-1}$, $P = 0.008$; leukocytes < $4.0.10^9 l^{-1}$ in 2/17 versus 7/16 patients respectively). At $t_2$ this difference was not observed.

Compared to $t_0$, a decreased leukocyte count was shown at $t_1$ in paired samples after both standard- and high-dose treatment ($P < 0.001$); this difference remained present at $t_2$ in both groups (Figure 1A).

### *Platelets*

Compared to the standard-dose group, platelet counts were lower in the high-dose group at $t_1$ (standard-dose: mean at $t_1$ $220.10^9 l^{-1}$, high-dose: mean $137.10^9 l^{-1}$, $P < 0.001$; platelets < $150.10^9 l^{-1}$ in 0/17 versus 11/16 patients respectively). At $t_2$ this difference was not observed.

Compared to $t_0$, a decreased leukocyte count was shown at $t_1$ in paired samples after both standard-and high-dose treatment ($P < 0.001$); this difference remained present at $t_2$ in both groups (Figure 1B).

### *Haemoglobin*

In the standard-dose group, haemoglobin values were not different at $t_0$, $t_1$ and $t_2$. Compared to the standard-dose group, haemoglobin was lower in the high-dose group at $t_1$ (standard-dose: mean at $t_1$ 8.0 mmol $l^{-1}$, high-dose: mean 6.7 mmol $l^{-1}$, $P < 0.001$; haemoglobin < 7.45 mmol $l^{-1}$ in 2/17 versus 14/16 patients respectively) as well as at $t_2$ ($P = 0.003$).

Compared to $t_0$, a decreased haemoglobin was observed at $t_1$ in paired samples after high-dose treatment ($P < 0.001$); this difference remained present at $t_2$ (Figure 1C).

### *MCV*

In the standard-dose group, MCV values were not different at $t_0$, $t_1$ and $t_2$. Compared to the standard-dose group, MCV values were increased in the high-dose group at $t_1$ (standard-dose: mean at $t_1$ 90.9 fl, high-dose: mean 97.9 fl, $P < 0.001$; MCV > 96 fl in 2/17 versus 10/16 patients respectively), but not at $t_2$.

Compared to $t_0$, an increased MCV value was observed at $t_1$ in paired samples after high-dose treatment ($P < 0.001$); this difference remained present at $t_2$ (Figure 1D).

### CD34+ cell number and haematological parameters

At $t_1$ in the high-dose group, the number of reinfused CD34+ cells (times $10^6$ per kg body weight) correlated positively with the number of leukocytes ($r = 0.63$; $P = 0.009$) and platelets ($r = 0.77$; $P < 0.001$), and negatively with MCV ($r = –0.6$, $P = 0.014$). No relation between haemoglobin and CD34+ cells was found. At $t_2$, no correlation between CD34+ cells and haematological parameters was observed.

### Telomere length and telomerase activity

TRF length (mean of all patients at $t_0$ 8.1 kb, SD 1.4) was in the same range as previously reported in cross-sectional studies

© 2001 Cancer Research Campaign

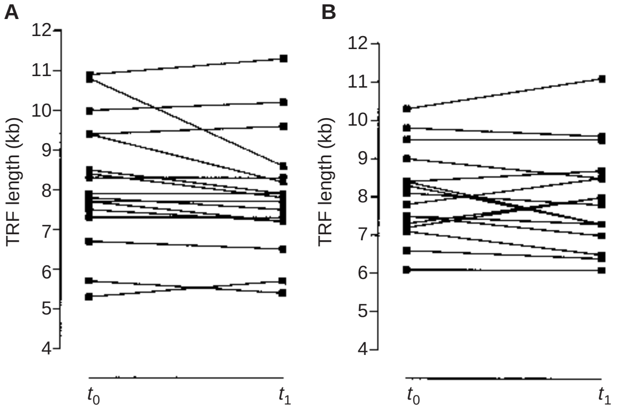

**Figure 2** Paired TRF samples. (**A**) X-axis: standard-dose group samples, at $t_0$ and $t_1$; Y-axis: TRF length (kilobase, kb). (**B**) X-axis: high-dose group samples, at $t_0$ and $t_1$; Y-axis: TRF length (kb)

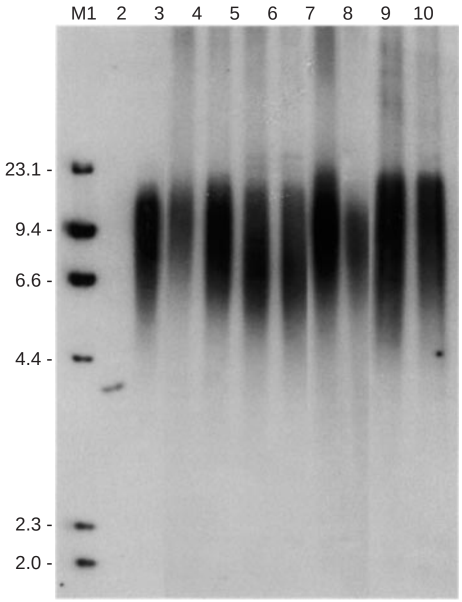

**Figure 3** Representative example of blot to measure TRF length. M: marker; lane 1: plasmid control; lane 2: leukocyte control healthy volunteer; lanes 3–10: paired patient samples; lanes 3 and 4: $t_0$ and $t_1$ sample, standard-dose treatment (Δ TRF length – 0.7 kb); lanes 5 and 6: $t_0$ and $t_1$ sample, standard-dose treatment (Δ TRF length 0 kb); lanes 7 and 8: $t_0$ and $t_1$ sample, high-dose treatment (Δ TRF length –1.1 kb); lanes 9 and 10: $t_0$ and $t_1$ sample, high-dose treatment (Δ TRF length + 0.3 kb)

(Akiyama et al, 1998; Ball et al, 1998; Wynn et al, 1998; Lee et al, 1999). As shown in Figure 2, TRF length decreased in 9 patients of each group when $t_0$ and $t_1$ samples were compared, and 4 patients from the standard-and 5 patients from the high-dose group showed a TRF length increase (mean Δ TRF length of both groups: –0.2 kb, SD 0.6; range Δ TRF length: standard-dose group: +0.4 to –2.2 kb; high-dose group +0.8 to –1.1 kb). Paired analysis of $t_0$ and $t_1$ samples showed overall no effect on TRF length of either treatment arm (standard-dose group: $P$ = 0.069; high-dose group: $P$ = 0.67) or of treatment in general (both groups together: $P$ = 0.148). A representative blot is shown in Figure 3. No difference in leukocyte differentiation was found when $t_0$ and $t_1$ samples were compared of both groups, and no difference between the groups was observed at $t_0$ or $t_1$.

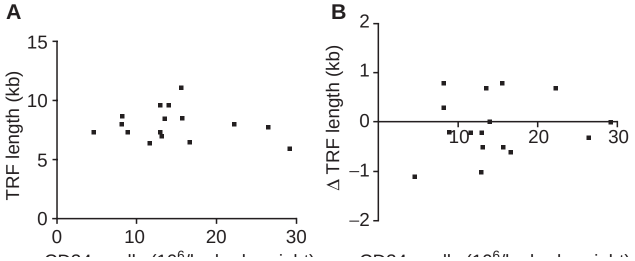

**Figure 4** TRF length and CD34 + cell numbers. On the X-axis, the number of reinfused CD34 + cells ($10^6$ per kg body weight) is indicated; on the Y-axis the following values are indicated: (**A**) TRF length (kb): measured value at $t_1$ after high-dose treatment, (**B**) Δ TRF length (kb): calculated difference between TRF values at $t_0$ and $t_1$ of high-dose treatment

In the high-dose group, no correlation between reinfused CD34+ cells and actual TRF length at $t_1$, or Δ TRF length could be observed (Figure 4).

Also the relation between haematological parameters haemoglobin, MCV, leukocyte and platelet counts at $t_1$ and $t_2$ and TRF length, or Δ TRF length was evaluated. No correlation between these haematological parameters and (Δ) TRF length could be observed.

In 9 patients from each group, paired leukocyte sample size also allowed measurement of telomerase activity at $t_0$ and $t_1$. This included the samples with maximum TRF length increase or decrease of both groups. Telomerase activity in all of these patient samples was below the reliable detection limit of 10 U (equivalent to 10 GLC4 cells) per $1.10^5$ leukocytes (Wisman et al, 1998), in both groups at $t_0$ and $t_1$. This activity level is comparable with telomerase activity found in leukocytes from healthy controls (Wolthers et al, 1999). Therefore, no strong up-regulation of telomerase activity was observed, also not in patients with increased TRF lengths after treatment.

### DISCUSSION

The perception that haematopoietic proliferative stress may accelerate the ageing of haematopoietic stem cells has gained interest, in view of the widespread use of haematopoietic stem cell transplantations for various clinical conditions. Evidence for accelerated telomere shortening after haematopoietic stem cell transplantations was found in a number of studies (Shapiro et al, 1996; Notaro et al, 1997; Akiyama et al, 1998; Ball et al, 1998; Wynn et al, 1998; Lee et al, 1999; Akiyama et al, 2000). Most data were derived from paediatric patients with haematological malignancies, and frequently mean TRF lengths after therapy were compared to mean TRF lengths of age-matched controls. However, mean TRF length of nucleated blood cells has been shown to be widely variable between these controls (Notaro et al, 1997; Akiyama et al, 1998; Ball et al, 1998; Wynn et al, 1998; Lee et al, 1999). Additionally, samples were drawn at a wide range of time after PBSCT, ranging from 1.6 months (Lee et al, 1999) to over 10 years (Akiyama et al, 1998; Wynn et al, 1999). Finally, as TRF dynamics were shown to be different in the various stages of life (Zeichner et al, 1999), predictive value for the adult setting may not automatically be assumed from these paediatric data. Therefore, we studied mean leukocyte TRF length in paired samples before and after treatment, in a group of high-risk breast cancer patients randomized to receive either

© 2001 Cancer Research Campaign adjuvant standard-dose chemotherapy, or adjuvant high-dose chemotherapy and PBSCT. These treatment modalities are frequently used for breast cancer (Antman et al, 1997), and their induction of haematopoietic stress and possible consequent effect on individual TRF length could be assessed. TRF length measurement in this study was performed based on the commonly used procedure by Harley et al (1990), and care was taken to standardize measurements. In analogy to the pioneering study by Wynn et al (1998), we chose unselected leukocytes to measure TRF length and telomerase activity in. In recent studies, it has been suggested that lymphocytes may have a larger TRF length than neutrophils (Wynn et al, 1999; Robertson et al, 2000). Although TRF length of T lymphocytes and neutrophils was shown to be equally affected by stem cell transplantation (Wynn et al, 1999), in case of a change in the relative proportions of these cells, it might be slightly more difficult to draw conclusions from overall leukocyte TRF length. However, in this study no difference in the leukocyte differentiations was found in either group before or after treatment, and TRF length in our leukocyte samples is therefore unlikely to be affected by such a difference. Furthermore, variable differences of TRF length of neutrophils and T lymphocytes have been reported, ranging from approximately 1 kb (Wynn et al, 1999) to none (Martens et al, 2000). In light of these data, we consider leukocytes, in line with Wynn et al (1998), sufficient for the purpose of this study.

Haematopoietic proliferative stress to achieve haematological reconstitution after treatment, was analysed by means of haematological parameters in peripheral blood, until 9 months after treatment. A clear negative effect on all haematological parameters was seen after high-dose treatment, and 9 months later still no recovery was made to the pre-treatment level. Even after standard-dose treatment, leukocyte and platelet counts were significantly affected for at least 9 months. A long-term impact of PBSCT on haematological reconstitution was observed in haematological malignancies (Barbui et al, 1996). Our data appear to support this in the solid tumour setting also, but data from longer follow-up periods are needed to confirm this. In line with previous studies (Faucher et al, 1996; Bernstein et al, 1998), we found that the number of reinfused CD34+ cells correlated with leukocyte and platelet numbers as well as MCV values, shortly after high-dose treatment.

Following the evident haematopoietic stress induced by both treatment arms (and PBSCT in particular), TRF length was clearly changed in individual patients. The majority of patients ($n$ = 9 in both arms) showed a TRF length decrease at $t_1$, but also remarkable TRF length increases were observed; no significant decrease due to either treatment was found in paired samples. The high-dose treatment scheme used in this study is classically combined with stem cell support in view of its profound myolotoxicity, causing prolonged life threatening marrow aplasia (Ayash et al, 1993; Antman et al, 1994). It is possible that in individual patients the lack of TRF length decrease due to treatment may be interpreted as a sign of insufficient treatment toxicity, as stem cells remaining in the patient after high-dose treatment will have an impact on the requirements to divide for haematopoietic reconstitution. In line with the presumed ablative nature of the treatment regimen in our study however, its profound impact on haematological parameters is clear. The maximum myelosuppression at $t_1$ and the (partial) haematological recovery at $t_2$, indicate haematopoietic proliferative stress at the time-point at which TRF length was measured (at $t_1$). Full recovery of haematological parameters after this high-dose treatment may actually take years (Nieboer et al, 2000), and the impact of this lengthy process on TRF length changes at later time-points than $t_1$ is currently being studied.

The detection of a distinct increase of TRF length in some patients was surprising. We hypothesized that up-regulation of telomerase activity in response to replicative stress might be responsible for this, in agreement with in vitro studies with purified CD34+ cells (Engelhardt et al, 1997; Yui et al, 1999). However, in our samples telomerase activity remained undetectable after treatment. In drawing conclusions from this, it should be considered that telomerase activity is a much more dynamic parameter than TRF length. Possibly, telomerase activity changes took place at other time-points than were measured in this study. Furthermore, in contrast to the comparable TRF length of leukocytes and CD34+ cells (Kronenwett et al, 1996), telomerase activity in purified CD34+ cells is likely higher than in terminally differentiated cells such as leukocytes (Engelhardt et al, 1997).

CD34+ cell numbers in our study were not related to (Δ) TRF length. Previously, it was assumed that if small numbers of CD34+ cells are reinfused, these cells may have to undergo more cell divisions than larger numbers, for a similar net haematopoietic effect (Notaro et al, 1997). However, no relationship was found between the degree of TRF length shortening and the number of reinfused CD34+ cells in recent studies (Lee et al, 1999; Wynn et al, 1999) and our data support this. Possibly, in vitro culturing of CD34+ cells may provide more insight into the balance between cell proliferation and the ability to upregulate telomerase activity in individuals, leading to (change of) telomere length in vivo. Disturbances in this balance may be related to haematological malignancies (Engelhardt et al, 2000). In this respect, the ability to measure TRF length in individual chromosomes or cells by means of flow cytometry (Rufer et al, 1998) or in situ hybridization (Martens et al, 2000) may be of interest. It remains conceivable that a rapid TRF decrease, predisposes for long-term effects such as secondary malignancies in individual patients. This has to be evaluated after a longer period of follow-up.

In conclusion, in this study we found that standard-and high-dose chemotherapy (in particular) negatively affect haematological reconstitution. Leukocyte TRF length was remarkably changed in individual patients after treatment, showing both decrease (in the majority of patients), as well as increase. Therefore, although no accelerated telomere loss was observed in general, TRF length was clearly affected following proliferative stress in this setting.

### REFERENCES

Akiyama M, Hoshi Y , Sakurai S, et al(1998) Changes of telomere length in children after hematopoietic stem cell transplantation. *Bone Marrow Transplant* **21**: 167–171

Akiyama M, Asai O, Kuraishi Y, et al (2000) Shortening of telomeres in recipients of both autologous and allogeneic hematopoietic stem cell transplantation. *Bone Marrow Transplant* **25**: 441–447

Antman K, Ayash L, Elias A, et al (1994) High-dose cyclophosphamide, thiotepa, and carboplatin with autologous marrow support in women with measurable advanced breast cancer responding to standard-dose therapy: analysis by age. *J Natl Cancer Inst Monogr* **16**: 91–94

Antman KH, Rowlings PA, Vaughan WP, et al (1997) High-dose chemotherapy with autologous hematopoietic stem-cell support for breast cancer in North America. *J Clin Oncol* **15**: 1870–1879

Ayash LJ, Elias A, Wheeler C, et al (1993) High-dose chemotherapy with autologous stem cell support for breast cancer: a review of the Dana-Farber Cancer Institute/Beth Israel Hospital experience. *J Hematother* **2**: 507–511

Ball SE, Gibson FM, Rizzo S, et al (1998) Progressive telomere shortening in aplastic anemia. *Blood* **91**: 3582–3592

 © 2001 Cancer Research Campaign

Barbui T, Cortelazzo S, Rossi A, et al (1996) Factors for rapid and sustained hematopoietic reconstitution by circulating progenitor-cell transplantation in non-Hodgkin's lymphoma. *Cancer Chemother Pharmacol* **38**: S110–S114

Bernstein SH, Nademanee AP, Vose JM, et al (1998) A multicenter study of platelet recovery and utilization in patients after myeloablative therapy and hematopoietic stem cell transplantation. *Blood* **91**: 3509–3517

De Vries EGE, ten Vergert EM, Mastenbroek CG, et al (1996) Breast cancer studies in the Netherlands. *Lancet* **348**: 407–408

Engelhardt M, Kumar R, Albanell J, et al (1997) Telomerase regulation, cell cycle, and telomere stability in primitive hematopoietic cells. *Blood* **90**: 182–193

Engelhardt M, Mackenzie K, Drullinsky P, et al (2000) Telomerase activity and telomere length in acute and chronic leukemia, pre- and post-ex vivo culture. *Cancer Res* **60**: 610–617

Faucher C, Le Corroler AG, Chabannon C, et al (1996) Autologous transplantation of blood stem cells mobilized with filgrastim alone in 93 patients with malignancies: the number of CD34+ cells reinfused is the only factor predicting both granulocyte and platelet recovery. *J Hematother* **5**: 663–670

Harley CB (1997) Human ageing and telomeres. In: *Telomeres and telomerase*. Ciba Foundation Symposium 211, p 129. Chichester, Wiley

Harley CB, Futcher AB and Greider CW (1990) Telomeres shorten during ageing of human fibroblasts. *Nature* **345**: 458–460

Hastie ND, Dempster M, Dunlop MG, et al (1990) Telomere reduction in human colorectal carcinoma and with ageing. *Nature* **346**: 866–868

Kronenwett R, Murea S, Haas R (1996) Telomere length of blood-derived mononuclear cells from cancer patients during G-CSF-enhanced marrow recovery. *Bone Marrow Transplant* S10–S14

Lansdorp PM (1995) Telomere length and proliferation potential of hematopoietic stem cells. *J Cell Sci* **108**: 1–6

Lee J, Kook H, Chung I , et al (1999) Telomere length changes in patients undergoing hematopoietic stem cell transplantation. *Bone Marrow Transplant* **24**: 411–415

Martens UM, Brass V, Engelhardt M, et al (2000) Measurement of telomere length in hematopoietic cells using in situ hybridization techniques. *Biochem Soc Trans* **28**: 245–250

Miller SA, Dykes DD and Polesky HF (1988) A simple salting out procedure for extracting DNA from human nucleated cells. *Nucleic Acids Res* **16**: 1215

Nieboer P, de Vries EGE, Mulder NH, et al (2000) Long-term hematologic recovery following high-dose chemotherapy with autologous bone marrow transplantation (ABMT) or peripheral blood stem-cell transplantation (PSCT) in patients with solid tumors. *Proc Amer Soc Clin Oncol* **19**: 193

Notaro R, Cimmino A, Tabarini D, et al (1997) In vivo telomere dynamics of human hematopoietic stem cells. *Proc Natl Acad Sci USA* **94**: 13782–13785

Ohyashiki K, Ohyashiki JH, Fujimura T, et al (1994) Telomere shortening in leukemic cells is related to their genetic alterations but not replicative capability. *Cancer Genet Cytogenet* **78**: 64–67

Ohyashiki JH, Iwama H, Yahata N, et al (1999) Telomere stability is frequently impaired in high-risk groups of patients with myelodysplastic syndromes. *Clin Cancer Res* **5**: 1155–1160

Robertson JD, Gale RE, Wynn RF, et al (2000) Dynamics of telomere shortening in neutrophils and T lymphocytes during ageing and the relationship to skewed X chromosome inactivation patterns. *Br J Haematology* **109**: 272–279

Royle NJ, Hill MC and Jeffreys AJ (1992) Isolation of telomere junction fragments by anchored polymerase chain reaction. *Proc R Soc London B Biol Sci* **247**: 57–61

Rudolph KL, Chang S, Lee HW, et al (1999) Longevity, stress response and cancer in ageing telomerase-deficient mice. *Cell* **96**: 701–712

Rufer N, Dragowska W and Thornbury G, et al (1998) Telomere length dynamics in human lymphocyte subpopulations measured by flow cytometry. *Nat Biotechnol* **16**: 743–747

Shapiro F, Engelhardt M, Ngok D, et al (1996) Telomere length shortening and recovery following high-dose chemotherapy with autologous peripheral stem cell rescue. *Proc Amer Soc Clin Oncol* 601A (abstr)

Vaziri H, Drakowska W, Allsopp R , et al (1994) Evidence for a mitotic clock in human hematopoietic stem cells: loss of telomeric DNA with age. *Proc Natl Acad Sci USA* **91**: 9857–9860

Wisman GBA, Hollema H, De Jong S, et al (1998) Telomerase activity as biomarker for (pre)neoplastic cervical disease in scrapings and frozen sections from patients with abnormal cervical smears. *J Clin Oncol* **16**: 2238–2245

Wolthers KC, Otto SA, Wisman GBA, et al (1999) Normal T-cell telomerase activity and upregulation in human immunodeficiency virus-1 infection. *Blood* **93**: 1011–1019

Wynn RF, Cross MA, Hatton C, et al (1998) Accelerated telomere shortening in young recipients of allogeneic bone-marrow transplants. *Lancet* **351**: 178–181

Wynn R, Thornley I Freedman M, et al (1999) Telomere shortening in leukocyte subsets of long-term survivors of allogeneic bone marrow transplantation. *Br J Haematol* **105**: 997–1001

Yui J, Chiu CP, Lansdorp PM, et al (1999) Telomerase activity in candidate stem cells from fetal liver and adult bone marrow. *Blood* **91**: 3255–3262

Zeichner SL, Palumbo P, Feng Y, et al (1999) Rapid telomere shortening in children. *Blood* **93**: 2824–2830

Zijlstra JG, de Vries EGE and Mulder NH (1987) Multifactorial drug resistance in an adriamycin-resistant human small cell lung cancer cell line. *Cancer Res* **47**: 1780–1784

© 2001 Cancer Research Campaign
